# Advances in the study of the correlation between balanoposthitis and skin microecology

**DOI:** 10.3389/fmicb.2025.1564675

**Published:** 2025-03-26

**Authors:** Mingyue Wang, Xinghua Gao, Li Zhang

**Affiliations:** ^1^Department of Dermatology, The First Hospital of China Medical University, Shenyang, China; ^2^Key Laboratory of Immunodermatology, Ministry of Education and NHC; National Joint Engineering Research Center for Theranostics of Immunological Skin Diseases, Shenyang, China

**Keywords:** balanoposthitis, skin microecology, glans penis, prepuce, treatment

## Abstract

Balanoposthitis is a common heterogeneous skin condition involving the glans penis and prepuce, and is seen from infancy to old age. Its predisposing factors are intertrigo, lengthy foreskin, localized irritation, and mucosal injury. The itching and skin inflammation at the glans penis seriously affects the quality of life of patients. As a result of advances in high-throughput sequencing technology, the prepuce microbial colonization patterns and glans penis are now known better. Previous studies have shown that an imbalance of flora can cause balanoposthitis. This article summarizes the progress of research on balanoposthitis and skin microecology, and provides a direction for the subsequent exploration of immunological mechanisms in balanoposthitis.

## 1 Introduction

Balanoposthitis (BP) is a common inflammatory condition involving the glans penis and prepuce in men, and is prevalent in people of all ages (English et al., 1997). As a male genitalia inflammation, BP is very common in urology, dermatology, and pediatrics. However, the diagnosis of BP is variable from one doctor to another across departments. From the 2013 European guideline for the management of BP to the 2022 European guideline to the latest updated Chinese expert consensus on the diagnosis and treatment of balanoposthitis, scholars have updated the definition of this disease in recent years (Edwards et al., 2014; Edwards et al., 2023; Zhang et al., 2024). The 2022 European Guideline (Edwards et al., 2023) define BP as inflammation caused by an infection, inflammatory dermatoses, or premalignant penile intraepithelial neoplasia. According to the Chinese expert consensus (Zhang et al., 2024), BP can be divided into infectious and non-infectious based on etiology. Infectious BP is caused by bacteria, fungi, and virus, and non-infectious BP is caused by an unknown cause with no obvious signs of infection (Zhang et al., 2024). BP has a wide range of causes and predisposing factors, and the treatment options for different causes vary greatly. Therefore, early prevention and identification of the cause are important. In recent years, more and more studies have shown that microbial communities are closely related to health and disease, and preventing or treating disease by regulating the microbiota has become a new strategy (Libertucci and Young, 2019; Waldman and Balskus, 2018). There have been several studies on the microbiome of semen in healthy and diseased states, but there are fewer studies on the microbiome of the genital mucosa of the glans and sub-prepuce (Gonçalves et al., 2022).

This article reviews the anatomy, etiology, pathogenesis, clinical manifestations, the relationship between BP and skin microecology, and the progress of therapeutic options. It further explores the role of microbiome alteration in the pathogenesis of BP under the condition of skin microecological imbalance as well as the future therapeutic prospects, aiming to provide theoretical reference bases for the future research on the immunological mechanism of glans penis and the choice of treatment.

## 2 Anatomical and histological characteristics

The male reproductive tract, similar to the visceral mucosa, is capable of generating an immune response, which is mediated by the cells that make up the reproductive immune system and by interactions with the local microenvironment, composed of sex hormones and microflora, which acts as a defense against the invasion of external pathogens (Nguyen et al., 2014). Learning the anatomical and histological characteristics of the mucosa at the glans prepuce is important for understanding the clinical manifestations and pathogenesis of BP. The human penis consists of four distinct regions: the prepuce (with a highly keratinized outer foreskin and a less keratinized inner foreskin), the glans (complex keratinized epithelium), the corpus cavernosum (complex non-keratinized epithelium), and the urethra (pseudocomplex non-keratinized epithelium). Squamous epithelium and a layer of dense connective tissue cover the glans penis, they are equivalent to the dermis of normal skin. The papillary layer of dermis is fused to the dense connective tissue and is attached to the tunica albuginea of the glans cavernosa (Halata and Munger, 1986). The prepuce is a continuation of the skin of the penile stem and covers the glans and urethra (Aridogan et al., 2011). The inner prepuce contains parietal sweat glands that secrete histone B, lysozyme, chymotrypsin, neutrophil elastase, cytokines, and androstenedione (Fleiss et al., 1998).

## 3 Clinical symptoms and differential diagnosis

BP is a heterogeneous condition that lacks clear diagnostic criteria, which makes it crucial to recognize the clinical manifestations and thus define the disease. However, different etiologies may result in different clinical manifestations and signs. Recognition of inflammatory lesions is a challenge for urologists, pediatricians, and dermatologists. An observational cross-sectional study found a significant correlation between different morphologies and the diagnosis of disease etiology (Jain et al., 2023). The common symptoms and signs of BP include itching, difficulty in retracting the foreskin, erythema, cracking, burning and pain (Edwards et al., 2023). Signs varied by causative agent. Candida glans had fissures with superficial pustules, and bacterial and herpetic glans had erosion/confluent moist erythema with sub-preputial discharge (Jain et al., 2023).

According to the Chinese expert consensus (Zhang et al., 2024), from the dermatologist’s point of view, the diagnosis of BP depends on clinical manifestations. Care needs to be taken to rule out some diseases, such as syphilis, genital herpes, fixed drug eruption, lichen sclerosus, lichen planus and pre-malignancy. Swab sampling and culture are used to identify the infectious causes. If no signs of infection are detected or there are no specific histological abnormalities, it is diagnosed as non - infectious balanoposthitis. Some systemic diseases that localize in the genital area or purely dermatologic conditions that cause inflammation are not categorized as part of BP. BP has been divided into two categories: infection-induced BP and non-infectious BP (Zhang et al., 2024). This is helpful in making it easier for physicians to clarify the diagnosis and definition of BP. Among the atypical manifestations of syphilis, some patients present with BP and penile edema (Rovira-López et al., 2015). In addition, a case report found that Follmann balanitis was an atypical form of primary cutaneous syphilis (Cubiró et al., 2020). Furthermore, ulcerative balanoposthitis may be the initial manifestation of acute promyelocytic leukemia (Steinbach et al., 1998). These findings from clinical work suggest that correct diagnosis and identification of the disease is essential for the treatment and prognosis. Bacterial and fungal cultures of swabs from lesions, serologic testing, dermoscopy, reflective confocal microscopy, bioposy are helpful in diagnosing and identifying the disease (Tønne et al., 2023).

## 4 Etiology and risk factors

The skin is exposed to the external environment and various microorganisms colonize the skin surface, *Propionibacterium acnes*, *Staphylococcus epidermidis* and *Corynebacterium* are common bacteria on the skin, and *Malassezia* is a common fungus on the skin (Byrd et al., 2018). In most cases, these microorganisms are harmless or even beneficial to the human body, adapting to the skin’s immune system, protecting the body from pathogens and breaking down natural metabolites (Pérez-Losada and Crandall, 2023). And when microecological imbalances lead to microbiota dysbiosis, the risk of infection increases (Smythe and Wilkinson, 2023). The skin can be categorized into moist, dry, and seborrheic areas based on their physicochemical properties. Wet sites (e.g., the anterior elbow fossa, groin, and popliteal fossa) provide large amounts of nutrients, such as salts, sterols, esters, and lipids, and are the dominant sites for the survival of *Staphylococcus* and *Corynebacterium*. In dry sites (e.g., palmaris minor and palmar aspect of the forearm) *Flavobacterium* spp. and *β-Amoeba* spp. predominate (Smythe and Wilkinson, 2023). Sebaceous glands in seborrheic areas (e.g., face, scalp, chest, and back) produce large amounts of oily sebum, which is the dominant site for *Propionibacterium acnes* and *Staphylococcus* (Grice et al., 2009). *Propionibacterium* spp., a genus of dermatobacteria (*Cutibacterium* spp.), as lipophilic anaerobes are widespread in sebaceous gland units of the hair follicle and produce lipases to convert triglycerides to short-chain fatty acids. In addition, *Staphylococcus* spp. are also capable of producing lipases to utilize lipid-rich substrates in these sites (Smythe and Wilkinson, 2023).

**Figure 1:**
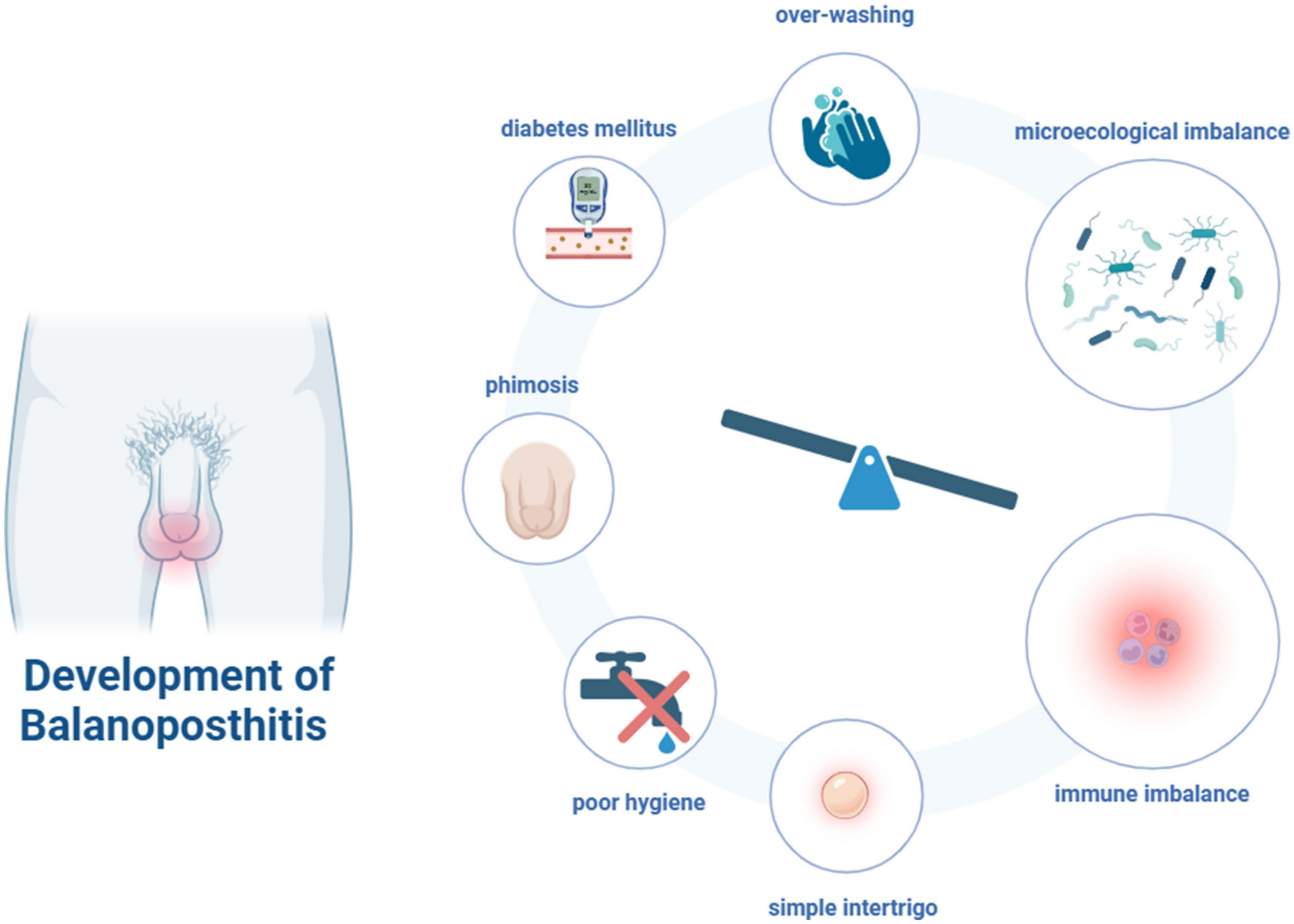
The risk factors and development of balanoposthitis (Figure created with BioRender.com).

BP can be triggered by a variety of factors, including phimosis, foreskin dysfunction localized irritation, poor hygiene, over-washing, and other underlying conditions (e.g., diabetes mellitus). In addition, many cases of BP are simple intertrigo. This is an inflammation occurring between two closely-apposed skin surfaces, often accompanied by the overgrowth of bacteria or fungi (Edwards et al., 2023). These factors are likely to affect the microecological imbalances in the male genital area thus causing infection and inflammation (Figure 1).

Physiologic phimosis is very common in children. With increasing age, physiologic phimosis will decrease (Hsieh et al., 2006). However, some patients may come to the doctor with BP caused by phimosis. This is due to changes in lipid content and moisture levels, which can cause local microecological imbalance and lead to inflammation. The *Malassezia* has been reported to be part of the microflora of the male genital region, as in other areas of human skin. The most common fungi in the penile microflora are *Malassezia sympodialis* and *Malassezia globosa*. The optimal growth conditions for *Malassezia* are acidic (pH = 5.5) and they are well adapted to dry but lipid-rich skin, however, when the glans is covered, the increase in humidity leads to an increase in pH, causing an imbalance in the flora leading to disease(Aridoğan et al., 2005; Mayser et al., 2001).

Diabetic patients have a higher risk of genitourinary infections (e.g., urinary tract infections, vaginitis and BP) than nondiabetic patients (La Vignera et al., 2019). An epidemiologic survey showed that among Brazilian men≥40 years of age who were screened for prostate cancer, BP accounted for 11.8% of penile lesions, having diabetes was considered an important risk factor (Romero et al., 2013). In addition, medications can cause genitourinary infections. Several studies have shown that sodium-glucose co-transporter-2 (SGLT2) inhibitors in medications for diabetes treatment increase the risk of urinary tract infections and genital fungal infections in routine clinical care (Dave et al., 2019; Liu et al., 2017; Lega et al., 2019). It is very common for diabetic patients to have BP with *Candida*. *Candida albicans* is an opportunistic human pathogen that causes severe mucosal infections in the host. Similar to *Malassezia*, the defining feature of *Candida albicans* pathogenesis is the transition from yeast to invasive filamentous hyphae. It has been shown that, similar to bacterial secretion of cytolytic proteins and peptide toxins that disrupt the skin’s immune barrier, the fungal cytolytic peptide toxins secreted by *Candida albicans* directly damage epithelial cell membranes and trigger signaling pathways that activate the epithelial immune response (Poulain, 2015). Several studies have shown that the common cause of infectious BP is infection by *Candida albicans*, *Streptococcus*, *Staphylococcus*, and *Malassezia* (Abdullah et al., 1992; Alsterholm et al., 2008).

Previous studies found the symptom of BP was very similar to that of bacterial vaginosis in women. BP is a multimicrobial synergistic etiology caused by a combination of *Gardnerella vaginalis* and anaerobic bacteria in the male lower genital tract and the female vagina (Burdge et al., 1986). It was found that BP is caused by several types of *Streptococcus pyogenes*, and genotype emm89 isolates play a role in BP infections in Japan (Hasegawa et al., 2017). Füzi et al. (1984) found that balanoposthitis was caused by *Streptococcus pyogenes* after sexual intercoursing. Interestingly, the main causative agent of BP in sexually active adolescents is *group B hemolytic streptococcus*, while few adolescents have *group A hemolytic streptococcus* infections (Norimatsu and Ohno, 2020). *Streptococcus pyogenes* is present in the human pharynx, and BP caused by *streptococcus pyogenes* may be transmitted by penile-oral intercourse (Minami et al., 2010). This suggests the need to classify *Streptococcus pyogenes* as one of the causative agents of BP and thus implement the appropriate sensitive antibiotic therapy. In addition, differentiation between different types of *hemolytic streptococci* is needed.

## 5 Infections and microecological imbalance

### 5.1 Interaction between microorganisms and the immune system and the impact of imbalance

Microorganisms can modulate innate and adaptive immune responses, and early interactions between the human microbiome and immune cells play a key role in the development of the immune system (Paller et al., 2019). Disturbances in the development and maturation of the microbiome early in life have an impact on the development of the infant’s immune system, leading to the development of atopic diseases such as asthma and atopic dermatitis (Peroni et al., 2020). The skin microbiota inhibits the colonization of pathogens such as *Staphylococcus aureus* and is an important component of epidermal barrier function. In contrast, when atopic dermatitis flares up, the normal microbiota is disrupted and the diversity of microorganisms on the skin is reduced (Paller et al., 2019; Geoghegan et al., 2018). *Staphylococcus aureus* colonizes the skin of patients with atopic dermatitis at significantly higher rates than *Staphylococcus epidermidis*. *Staphylococcus aureus* compromises the skin barrier by down-regulating the terminal differentiation of epidermal proteins (e.g., polysilk proteins and dystrophin), promoting dermal protease activity, and internalizing into keratinocytes and inducing an imbalanced Th1/Th2 adaptive immune response via Langerhans cells (Peroni et al., 2020; Iwamoto et al., 2019).

### 5.2 Characteristics of microorganisms in the male genital area and their correlation with related infections

While traditional microbial culture techniques are only capable of detecting dominant strains adapted to specific skin sites, more modern genomic testing techniques offer the possibility of detecting the full spectrum of strain distribution. With the advent of high-throughput sequencing technologies such as macro-genome sequencing, whole-genome birdshot, and next-generation sequencing (NGS) technologies, the pattern of bacterial colonization in the anatomical compartments of the male genital tract is becoming better understood, and the male genital tract is an ecosystem with lower bacterial abundance but a relatively diverse bacterial community, compared to other parts of the human body (Zuber et al., 2023). Bacterial genera of the male genital mucosa include *Prevotella* spp., *Finegoldia* spp., *Peptoniphilus* spp., *Staphylococcus* spp., *Corynebacterium* spp. and *Anaerococcus* spp. The microbial composition of the male genital mucosa was similar to that of the adjacent anatomical sites and correlated with sexual intercourse (Gonçalves et al., 2022). Zozaya et al. (2016) found that the flora on the surface of men’s penises was very similar to the flora of their female partners with bacterial vaginitis, but very different from the flora of other women with bacterial vaginitis, suggesting that transmission of bacterial vaginitis-associated flora through sexual intercourse is common (Zozaya et al., 2016). Bacterial dysbiosis, circumcision length and impaired physical barrier are the causative factors of BP. Li et al. (2021) found that *Staphylococcus warneri* and *Prevotella bivia* were the common bacteria found in the genital mucosal area of patients with BP and these two bacteria were positively correlated with the severity of the condition. In addition, it was found that lifestyle had an impact on the distribution of bacteria, such as *Staphylococcus warneri* was predominant in those who used condoms, and *Prevotella bivia* was predominant in those who were not sexually active (Li et al., 2021).

## 6 Immunity

Based on the understanding of the complex interactions between microorganisms and infections in the male genital area, it is necessary to deeply explore the role of the immune system in maintaining the balance of this region and the mechanisms of disease occurrence when the immune system is imbalanced.

Different regions of the penis have different immune cell compositions and phenotypes. For example, large numbers of B and T cells are present in the glans, whereas NK cells are predominantly present in the corpus cavernosum. Unlike other penile regions, the mucosal immune system of the foreskin has been intensively studied. The lamina propria and epithelium of the foreskin selectively contain macrophages and CD1a + HLA-DR+ LCs, respectively (Sennepin et al., 2017). In addition, the foreskin contains dendritic cells, CD4 + T/CD8+ T cells and a large number of memory T cells, which play an important role in intrinsic and adaptive immunity. The foreskin epithelium is also protected by mucins (MUC1 and MUC4) and expresses Toll-like receptors (TLR5, TLR4, and TLR3, among others), which gives the tissue anti-infective potential (Sennepin et al., 2017; Brotman et al., 2014). Notably, the foreskin is coexisting with CD3+ CD4-/CD8- T cells capable of producing CD3+ CD4-/CD8- T cells at twice the ratio of their blood counterparts, and this double-negative T cells are a potential subpopulation of cells capable of performing a CD4+ T cell-like cofactor function in the event of CD4+ T cell depletion, and thus they have an important role to play in the prevention of SIV and HIV immunopathogenesis (Milush et al., 2011). Currently, most studies have focused on the mechanism of immune response at the glans penis site triggered by SIV or HIV pathogenic conditions, whereas the proportion of immune cells constituting the BP site and the mechanism of action in the disease state need to be further investigated. The establishment of a disease model for BP is expected to provide further insight into the pathogenesis. However, there is currently no model for BP. The anatomy of the genitalia of mice is similar to that of humans (Cunha et al., 2020). We hypothesize that altering the microecology in the genital area of mice can achieve the construction of a model for the BP, but the survival environment of the dominant flora is needed to be clarified. At the same time, the preparation of an environment for *anaerobic bacteria* was difficult and challenging from our previous work. In the future, more work still needs to be put into studying BP modeling.

## 7 Therapeutic options

Regarding the treatment of BP, most treatments for balanoposthitis are empirical, the mainstays are oral or topical medications, retraction of the prepuce, as well as circumcision. For penile glans infections caused by fungi, treatment options include topical or oral azoles such as clotrimazole, miconazole, fluconazole, and sertaconazole (Lisboa et al., 2009). When there are indications of bacterial co-infection, the appropriate use of broad-spectrum antibiotics to fully cover the causative organisms may contribute to rapid remission during the disease management process.

For acute BP, which is often accompanied by oozing, and wet compress therapy is highly effective. The efficacy of copper sulfate, zinc sulfate and alum was superior to that of saline solution (Gonzalvo et al., 2015). This is similar to the use of saline or boric acid solution in acute eczema flare-ups to astringe the exudate and reduce redness and oozing. For children with BP, the questionnaire showed that most people adopted bathing, topical gel, or topical antibiotics, either singly or in combination (Zundel and Ellerkamp, 2024). Perhaps it is more acceptable for doctors to use a simpler, non-invasive approach in conjunction with parental wishes. More investigations are needed in the future to evaluate non-invasive means of treating BP.

Topical steroid therapy is often used to reduce the phimosis. The researchers found that in the treatment of grade 4–5 phimosis, 1% hydrocortisone cream used in conjunction with 0.1% triamcinolone cream resulted in a therapeutic response for up to 12 weeks (Chamberlin et al., 2019). The meta-analysis indicated that the efficacy of moderate-to-low-potency topical corticosteroids was comparable to that of high potency topical corticosteroids in the treatment of phimosis (Shanmugham et al., 2024). Effective treatment of phimosis can slow the progression of the disease and reduce the incidence of BP. It is worth noting that there are certain risks associated with the topical steroids, such as causing microecological imbalance, skin atrophy, and suppression of the immune response (Coondoo et al., 2014).

For non-infectious BP, in clinical practice, simply retracting the prepuce can cure more than 90% of balanitis, but attention should be paid to the risk of paraphimosis, circumcision is a last resort. However, there is currently no literature support for this experience. Male circumcision involves the surgical removal of some or all of the foreskin from the penis (American Academy of Pediatrics Task Force on Circumcision, 2012). Male circumcision affects the penile microbiome, Liu et al. found that total bacterial load and microbiota diversity were significantly reduced after circumcision, with a significant decrease in the colonization rate and abundance of anaerobic flora. Although aerobic flora increased after circumcision, it was not significant (Liu et al., 2013). The results of the meta-analysis showed that the incidence of BP after circumcision was 68% lower than that of uncircumcised men, and the incidence of BP secondary to cancer decreased by 3.8 times, which means that early circumcision can effectively reduce the incidence of BP (Morris and Krieger, 2017). Epidemiologic studies have shown that circumcision affects the type and incidence of genital dermatoses and that most cases of inflammatory dermatoses are diagnosed in uncircumcised men (Elakis and Hall, 2017). Previous study has shown that circumcision in infancy reduces the risk of HIV and other sexually transmitted infections (Morris et al., 2022). It is also an important intervention in controlling the prevalence of HIV (Farley et al., 2020). A survey to assess the rate of fungal colonization at the penis in circumcised patients showed that yeast was found on the glans in 55 (22.4%) of 245 circumcised pediatric patients examined. Of these, *Malassezia* was detected in 17 (30.9%), *Candida* in 36 (65.5%), *Malassezia* and *Candida* in 1 (1.8%), and *Saccharomyces cerevisiae* in 1 (1.8%). Among lipophilic yeasts, *Malassezia furfur* was the most common (66.7%), followed by *Malassezia globosa* (11.1%), and among non-lipophilic yeasts, *Candida albicans* was the most common (46.0%) (Aridoğan et al., 2005). Another study comparing the rate of yeast colonization in the penile glans and foreskin before and 1 month after circumcision found that the rate of yeast colonization decreased from 11.7 to 1.3% after circumcision, with *Candida albicans* predominating in the penile glans and foreskin (50%), followed by *Malassezia furfur* (40%) and *Malassezia ensiformis* (10%). The researchers hypothesized that the change in skin pH caused by circumcision plays a key role in the colonization of the glans by *Malassezia* spp. (Aridogan et al., 2009).

As a non-invasive technique, photodynamic inactivation (PDI) becomes an alternative to conventional treatment (Kawczyk-Krupka et al., 2007). A recent study demonstrated that methylene blue (MB) and silver prismatic nanoplatelets (AgNPrs) conjugates in PDI have a significant inactivating effect on *Candida albicans*. This study optimizes PDI and provides a new strategy for the treatment of drug-resistant microorganisms screened out in patients with BP (Rodrigues et al., 2024). In conclusion, the means of treating glans penis are abundant, and the doctor needs to take into account the indications as well as the wishes of the patient or the parents to realize a better treatment.

## 8 Discussion and conclusion

BP is a common skin condition that affects the male genital area and is more frequently seen in the uncircumcised group (Tønne et al., 2023). Pain, itching and swelling of the foreskin and glans seriously affect the quality of life of the patient (Tønne et al., 2023). This review reinterprets the relationship between the skin microecology of the glans penis and prepuce and balanoposthitis based on the expert consensus on balanoposthitis (Zhang et al., 2024). In diagnosing BP, Chinese expert consensus has simplified its diagnosis and definition by dividing it into two categories, infectious BP and non-infectious BP, believing this will be more helpful for guiding physicians in their clinical work (Zhang et al., 2024). In addition, this review summarizes the risk factors of BP, the impact of treatment methods on the microbial flora in the glans penis and prepuce area, and the possible immune mechanisms that may cause BP. These findings suggest that changes in the microbial flora may affect the microecological balance in the glans penis and prepuce area, thereby triggering BP. Currently, the research on BP mainly focuses on the clinical level, such as the clinical symptoms, diagnosis, and treatment methods of the disease. Restricted by the current research status, this review has limitations in deeply exploring the immunological mechanisms of the disease occurrence. It fails to comprehensively analyze how the imbalance of the microbial flora specifically triggers an immune response and then leads to the detailed process of BP. In addition, the onset of BP is the result of the combined action of multiple factors. However, this review mostly elaborates on the association between each factor and the condition separately, lacking an in - depth exploration of the interactions among these factors.

The relationships between skin diseases such as atopic dermatitis and acne and the skin microecology have been extensively studied, and the corresponding treatment methods have been widely applied at the clinical level (Mohammad et al., 2024; Huang et al., 2023). However, the research on the microecology of BP is still in its initial stage, and both the depth and breadth of this research need to be expanded. Previous systematic review has identified the composition of microbial communities in different anatomical sites of the genital mucosa of healthy and diseased men, and proposed that changes in host - genital microbiome interactions may be associated with disease development (Gonçalves et al., 2022). However, due to the limitations of these studies, only the bacterial community through 16S rRNA gene amplicon sequencing was analyzed, and it is necessary to investigate the fungal community, viral community and parasite community in the male genital mucosa (Gonçalves et al., 2022). This will contribute to a more comprehensive understanding of the microecological structure of the male genital mucosa and provide a theoretical basis for the mechanism research of BP.

High-throughput metagenomics detection technology makes it possible to have a more comprehensive understanding of the microecological distribution in the glans penis and prepuce area. Li et al. found that, compared with healthy individuals with normal prepuce, patients with BP had a higher abundance of *Finegoldia magna* and Bradyrhizobium at the genus level (Li et al., 2021). At the species level, the abundances of *Staphylococcus warneri* and *Prevotella bivia* in patients with BP were significantly higher than those in healthy individuals (Li et al., 2021). However, the numbers of healthy people and patients with BP included in this study were relatively small. In the future, it is necessary to further expand the sample size and conduct in-depth research on the mechanisms of action of *Staphylococcus warneri* and *Prevotella bivia* in balanoposthitis.

The genital structure of male mice is relatively similar to that of humans. However, currently, there is no report of a successfully established mouse model of BP internationally. The models that have been successfully established mainly focus on penile cancer (Medeiros-Fonseca et al., 2021). In specific environmental bacterial microbial communities, there is a strong correlation between microbial metabolites and the gene content of the community. The basis for symbiosis among different microorganisms lies in the relationship of resource consumption and sharing among them (Gowda et al., 2022). By simulating the microenvironment of BP, clarifying the distribution of dominant fungal and bacterial species in the glans penis and prepuce area and their interactions may provide ideas for establishing a model of balanoposthitis and further in - depth research on the pathogenesis.

Understanding the microbiome and immunological mechanisms of glans penis can help to develop personalized treatment plans in the clinic and provide more possibilities for early intervention of BP. Circumcision and simply retracting the prepuce can cause dryness and keratinization in the preputial environment, creating a micro - environment that is less conducive to the proliferation of bacteria or fungi. Therefore, clarify the impact of microecology on the BP is needed. In the clinical field, with the development of technology, doctors can rapidly diagnose BP through dermoscopy. In the research field, high-throughput sequencing can further clarify the changes in the flora of the genital area. Therefore, it is necessary to study BP in depth in the future to clarify the pathogenesis of BP and the effects of flora interactions on men in healthy and diseased states. Further research into BP is still needed to provide more informed clinical decisions in the future.
